# An injectable *in situ* gel with cubic and hexagonal nanostructures for local treatment of chronic periodontitis

**DOI:** 10.1080/10717544.2017.1359703

**Published:** 2017-08-17

**Authors:** Liling Mei, Xintian Huang, Yecheng Xie, Jintian Chen, Ying Huang, Bei Wang, Hui Wang, Xin Pan, Chuanbin Wu

**Affiliations:** School of Pharmaceutical Sciences, Sun Yat-sen University, Guangzhou, China

**Keywords:** Inverse liquid crystalline, cubic phase, hexagonal phase, *in situ* gel, anti-bacterial treatment

## Abstract

Periodontitis is a chronic bacterial infection, and its effective treatment is dependent on the retention of antibiotics of effective concentrations at the periodontal pockets. In this study, a solution–gel based inverse lyotropic liquid crystalline (LLC) system was explored to deliver metronidazole to the periodontal pockets for local treatment of periodontitis. It was found that the metronidazole-loaded LLC precursor spontaneously transformed into gel in the presence of water in the oral cavity. The low viscosity of the precursor would allow its penetration to the rather difficult to reach infection sites, while the adhesiveness and crystalline nanostructures (inverse bicontinuous cubic Pn3m phase and inverse hexagonal phase) of the formed gel would permit its firm adhesion to the periodontal pockets. The LLC system provided sustained drug release over one week *in vitro*. Results from *in vivo* study using a rabbit periodontitis model showed that the LLC system was able to maintain the metronidazole concentrations in the periodontal pockets above the minimum inhibition concentration for over 10 days without detectable drug concentration in the blood. Owing to the spontaneous solution–gel transition in the periodontal pockets and unique liquid crystalline nanostructures, the LLC *in situ* gel provided effective treatment of periodontitis for a prolonged period of time with reduced systematic side effects, compared to metronidazole suspension which was effective for 24 h with detectable metronidazole concentrations in the blood after 6 h.

## 1. Introduction

Periodontitis is a bacterial infection highly prevalent in adults with 47% of adults in the US suffering from it, and it gets worse for adults older than 65 years with 64% prevalence of the population (Eke et al., 2012). Periodontitis causes inflammation and tissue destruction by microorganism colonization, leading to the formation of periodontal pockets (He et al., 2015; Joshi et al., 2016; Phaechamud et al., 2016). Without effective therapy, it could result in progressive loosening and subsequent loss of teeth.

The treatment of this chronic infection requires antibacterial medication. However, it is difficult to achieve effective drug concentrations at the microbial infection sites, periodontal pockets, by the commonly used oral administration of antibiotics. In addition, the distribution of drug in other tissues and organs associated with frequent oral administration would cause both side effects and antibiotic resistance, especially following long-term therapy (Barat et al., 2006; Reise et al., 2012). The local depot system which provides sustained drug release at the sites of action and therefore beneficial in maintaining effective drug concentrations and reducing systematic side effects, has been proposed for the treatment of periodontitis (Do et al., 2014, 2015; Lee et al., 2016).

However, effective delivery of antibiotics to the periodontal pockets is still met with various challenges and more effective systems are needed (Hau et al., 2014), recognizing that the periodontal pockets are deep and small spaces around infected teeth (Dabhi et al., 2010). They are difficult for drug to reach and retain (Srivastava et al., 2016).

A desirable intra-pocket drug delivery system should be a low viscosity fluid for good penetration into the sites of infection. The system should also possess good adhesiveness for retention in the periodontal pockets. *In situ* gel appears to be a good candidate to meet the requirements of both low viscosity and superior adhesiveness (Nasra et al., 2017). It is administrated in the form of precursor which is transformed into a gel at the sites of action. The phase transition from solution to gel is the key to the success of the *in situ* gels for the treatment of periodontitis. Several *in situ* gels, such as pH-dependent, light-respondent (Nguyen & Hiorth, 2015), and temperature-sensitive gels (Kelly et al., 2004), have demonstrated potentials in achieving solution–gel transition in the periodontal pockets for adhesion and retention. However, they failed to provide prompt enough solution–gel phase transition because the triggers of solution–gel transition, such as the pH shift for the pH-dependent systems and the temperature variation for the temperature-sensitive carriers, are either infeasible or insensitive in the periodontal pockets. Extensive adhesion and retention at the sites of action are barely achieved by these *in situ* gels.

Promisingly, the solution–gel transition trigger of inverse lyotropic liquid crystalline (LLC) system is simply the addition of water (Gong et al., 2011). It can easily absorb adequate water in the gingival crevice fluid (GCF) to transform into an *in situ* gel which would attach to the periodontal tissues. The LLC could be an excellent candidate as an intra-pocket drug delivery system for the treatment of periodontitis owing to its unique properties of solution–gel phase transition, unique nanostructures, good adhesiveness, and gel strength (Scriven, 1976; Sagalowicz et al., 2006).

Desirable properties of an intra-pocket drug delivery system for the treatment of periodontitis include: sustained drug release for long-term antimicrobial effect, low viscosity for administration, and good adhesion for retention. Lack of any of these properties may lead to failure in effective drug delivery. There have been increased interests in optimizing these properties to identify effective intra-pocket depot carrier systems (Da Rocha et al., 2015; Chen et al., 2016; Joshi et al., 2016). The LLC system presented in this work was found to possess the needed low viscosity, sensitive solution–gel phase transition, favorable mechanical properties, and sustained drug release behavior.

## 2. Materials and methods

### 2.1. Materials

*Agents*: Glycerol monooleate (GMO, DIMODAN^®^MO/D KOSHER) was a present from Danisco Cultor (Brabrand, Denmark). *N*-methyl pyrrolidone (NMP) was purchased from Guangfu Smart Chemical Research Institute (Tianjin, China). Medium chain triglyceride (MCT) was purchased from Gracia Chemical Technology Co., Ltd. (Chengdu, China). Metronidazole (MTZ) was obtained from Huanggang SaiKang Pharmacy Co., Ltd. (purity ≥99.0%, Huanggang, China). MTZ tablets were produced by Kangmei Pharmaceutical Co., Ltd. (Jieyang, China). Ligature suture (3–0) was purchased from Johnson & Johnson Medical Ltd. (Shanghai, China). *Porphyromonas gingivalis* lipopolysaccharide (LPS-PG) and pentobarbital sodium were obtained from Invivogen (San Diego, CA) and Sigma (Shanghai, China), respectively. All other chemicals were of analytical grade and used as received.

*Animals*: New Zealand rabbits, 10 weeks old, weighing approximately 3.0 kg, were purchased from the Laboratory Animal Center of Sun Yat-sen University (Guangzhou, China). Studies were conducted in accordance with guidelines and procedures approved by the Institutional Authority for Laboratory Animal Care and Ethics Committee of Sun Yat-sen University.

### 2.2. Preparation of the LLC precursor

The molten GMO was dissolved in NMP in the weight ratios from 1:9 to 9:1with stirring at 2000 rpm for 30 min to obtain GMO–NMP binary precursors (Table 1). Various amounts of MCT ranged from 0 to 15% (w/w) were added to prepare GMO–NMP–MCT ternary precursor preparations. MTZ-loaded formulations were also prepared by adding definite amount of MTZ.

**Table 1.** Formulation composition of GMO/NMP binary precursors (BPs, containing 25 wt% MTZ), gelation time, and sustained release time of corresponding *in situ* gels at 37 ± 0.5 °C (*n* = 3).

Formulations	GMO:NMP (w/w)	Gelation time (s)	Sustained release time (h)
BP-1	1:9	85.7 ± 4.7	2.2 ± 0.1
BP-2	2:8	50.2 ± 3.2	2.6 ± 0.7
BP-3	3:7	9.4 ± 2.5	4.3 ± 1.5
BP-4	4:6	8.7 ± 1.3	6.2 ± 2.4
BP-5	5:5	8.1 ± 1.1	6.7 ± 2.9
BP-6	6:4	6.3 ± 0.6	8.7 ± 2.7
BP-7	7:3	3.7 ± 0.6	48.2 ± 4.2
BP-8	8:2	2.4 ± 0.3	72.9 ± 4.5
BP-9^a^	9:1	ND	ND

GMO: glycerol monooleate; NMP: N-methyl pyrrolidone; MTZ: metronidazole.

^a^ GMO crystallization was observed in the BP-9 formulation and thus the gelation time and sustained release was not determined (ND).

The *in situ* gels for further studies were obtained by exposure of the LLC precursors to excess water, followed by static phase equilibration over one week and then centrifugation at 300*g* to remove excess water and air bubbles.

### 2.3. Viscosity test of the LLC precursor

Viscosity of the 25% MTZ-loaded LLC precursor was determined by a Brookfield rotational rheometer with a number 18 spindle at 25 ± 0.5 °C. The spindle rotating rate was increased by 5 rpm until the torque reached 100%.

### 2.4. Gelation time test of the LLC precursor

The gelation time of the phase transition is the time needed for the precursor transforming into gel upon the addition of the precursor into excess water. A 0.1 g precursor containing 25% w/w MTZ was injected into 25 mL preheated pH 7.4 PBS at 37 ± 0.5 °C through a modified pipette tip. The time upon the precursor contacting with aqueous dissolution medium till it completely transforming into opaque gel was recorded as gelation time.

### 2.5. Phase investigation and nanostructure analysis

The liquid crystalline phase of the LLC gel was observed by cross-polarized microscopy (CPM, MP41, Mshot, China) with a heat stage (KER3100-08S Mshot, China) thermostatically at 37 ± 0.5 °C or heated from 25 to 40 °C at a rate of 2 °C/min.

Further phase identification and structure analysis of crystalline cell nanostructure were performed with small angle X-ray scattering (SAXS, SAXSess, Anton Paar, UK) at 37 ± 0.5 °C. The gel sample was wrapped in aluminum foil and fixed in a sample holder. The wavelength of the X-ray radiated by a Cu Kα emitter under 50 kV and 40 mA was 0.1542 nm. The scattering factor *q* of Bragg peaks was set from 0.04 to 4.00 nm^−1^. The specific parameters of crystalline cell were calculated by the following equations (Caboi et al., 2001):

(1) q=(4πsinθ)/λ

(2) d=2π/q

(3) $$a=\left(h^{\text{2}}+k^{\text{2}}+l^{\text{2}}\right)\text{1}/\text{2}d$$

where 2*θ* is the scattering angle and *λ* is the wavelength of 0.1542 nm, *d* is the crystalline inter-planar space of liquid crystalline phase, *a* is crystalline lattice parameter which indicates the size of water channels in the liquid crystalline cell nanostructure, *h*, *k*, and *l* are Ptolemy indexes and have no dimension.

### 2.6. Mechanical properties of the LLC gel

#### 2.6.1. Adhesiveness and gel strength of the LLC gel

Adhesiveness and gel strength of the LLC gel were tested by a texture analyzer (TA.XT. Plus, Surrey, UK). A specific probe P 0.5 was impelled into the prepared gel sample vertically at a speed of 0.5 mm/s until 4 mm deep into the gel. Then, the probe was withdrawn gradually at a speed of 10 mm/s. The probe displacement and applied force as a function of time were recorded during the measurement. The adhesiveness and gel strength were defined as the maximum force values in the negative and positive directions of the measured force, respectively.

#### 2.6.2. Viscoelasticity of the LLC gel

Viscoelasticity of the LLC gel was determined using a rotational rheometer (Kinexus Lab+, Malvern, UK) equipped with a CP 1/60 cone-plate geometry. The measurement was conducted in a strain-controlled mode (sample gap of 0.50 mm) and in a frequency range of 0.01–100 rad s^−1^ at 37 ± 0.5 °C.

### 2.7. *In vitro* drug release

The *in vitro* drug release test was conducted in a thermostatic shaker (THZ-82BA, Jintan, China) at 37 ± 0.5 °C, 100 rpm. Approximately 0.2 g MTZ-loaded precursor was accurately weighed and injected into 25 mL pH 7.4 PBS. Samples were withdrawn and replaced with equivalent amount of fresh medium at predetermined time intervals. The collected samples were filtered with 0.22 μm filter prior to high performance liquid chromatography analysis (detailed method shown in the supporting information). The time needed for complete drug release was recorded as sustained release time.

The structure change and phase transition of the gels were observed by CPM with the same release condition in different experiment.

### 2.8. *In vivo* evaluation

To evaluate the efficacy and side effects of the LLC *in situ* gel, a rabbit periodontitis model was established by ligature (3–0 suture) in combination with inoculation of LPS-PG (Hasturk et al., 2006; Van Dyke, 2008) following anesthesia (ear intravenous injection of 3% w/w pentobarbital sodium at a dosage of 30 mg kg^−1^). Ten rabbits were randomized into two groups, and subject to local administration of the MTZ-loaded LLC precursor (containing 52.6% GMO, 13.2% NMP, 4.2% MCT, and 30% MCT, w/w) and MTZ suspension prepared from MTZ tablets, at 30 mg kg^−1^ MTZ, respectively.

Samples of GCF and blood were collected prior to administration and at predetermined time intervals post-administration by sterile orthodontic point method and auricular vein sampling method, respectively. The concentrations of MTZ in the GCF and blood samples were analyzed by high performance liquid chromatography method. The detailed methods of sampling and sample analysis can be found in the supporting information.

### 2.9. Statistics analysis

The data were expressed as mean ± S.D. Student *t*-test (SPSS 17.0, Chicago, IL) was employed for statistical analysis. *p* < .05 was set as the significance level.

## 3. Results and discussion

### 3.1. Development of GMO–NMP–MCT ternary system

The low viscosity precursor systems consisted of LLC material, GMO, and solvent NMP. GMO, a food additive approved by the Food and Drug Administration, is able to spontaneously form various crystalline types of LLC in the presence of different amounts of water (Mei et al., 2017). It is biodegradable and the degraded products, glyceride, and oleic acid, are safe endogenous substances (Warren et al., 2011; Wadsater et al., 2014). NMP was used to dissolve GMO. It is safe (Li et al., 2005; Saw et al., 2007; Qin et al., 2016) and capable of improving the loading content of MTZ (Figure S1).

The LLC precursor spontaneously transformed to *in situ* gel in excess water (Video S1). After injection of the LLC precursor into the periodontal pockets, the water in the GCF penetrates into the precursor and *in situ* gel is formed. Previous studies by Yaghmur et al. also demonstrated that dynamical structural transition and fast formation of liquid crystalline gel were observed upon exposure of the precursor to biological environment (Yaghmur et al., 2011). Short gelation time for the solution–gel phase transition is important for the prompt attachment of the gel to the teeth. Reduced gelation time is desired to minimize the loss of intra-pocket carrier and thus improve the accuracy of dosing. It was found that the gelation time was reduced by increasing the GMO/NMP ratio (w/w). However, it was found that the amount of NMP was insufficient to dissolve GMO when its content was lower than 10% (w/w). Therefore, the highest GMO/NMP ratio in the preparation of LLC precursors was kept at 8/2, and its gelation time was found to be 2.4 s and sustained release time for complete drug release was found to be 72.9 h as shown in Table 1.

Rapid gelation would reduce the initial burst release as the formation of gel would retard the release of drug. The reduction of gelation time from 85.7 to 2.4 s, caused by increasing GMO content from 10% to 80% w/w, dramatically increased the sustained release time, which was prolonged from 2.2 to 72.9 h (Table 1).

The maximum sustained drug release time (72.9 h) from the LLC gel transforming from GMO–NMP binary precursor was insufficient, and it is favorable for intra-pocket depot system to offer extended drug release over one week for effective periodontitis treatment (Schwach-Abdellaoui et al., 2000; Bal et al., 2015). MCT was added to the LLC precursor to modify the drug release rate (Yaghmur et al., 2012a).

To define the MCT content, the liquid crystalline phase transition induced by addition of MCT (0–15%, w/w) was investigated (Figure S2). Increasing addition of MCT induced a cubic (0–2% MCT)-hexagonal (3–13% MCT)-inverse micellar phase (14–15% MCT) transition of the LLC gels at 37 ± 0.5 °C. The phase transition was consistent with previous reports (Yaghmur et al., 2005, 2006, 2012b; Wibroe et al., 2015). In order to obtain cubic and hexagonal phase, which were reported to retard the drug release *via* incorporation of drug into their highly ordered complex nanostructure networks (Scriven, 1976; Mezzenga et al., 2005; Yaghmur et al., 2012a), 0–10 wt% MCT was added into the precursors for further studies.

### 3.2. Phase identification of the LLC gel transforming from MTZ-loaded GMO–NMP–MCT ternary precursor

**Figure 1.**
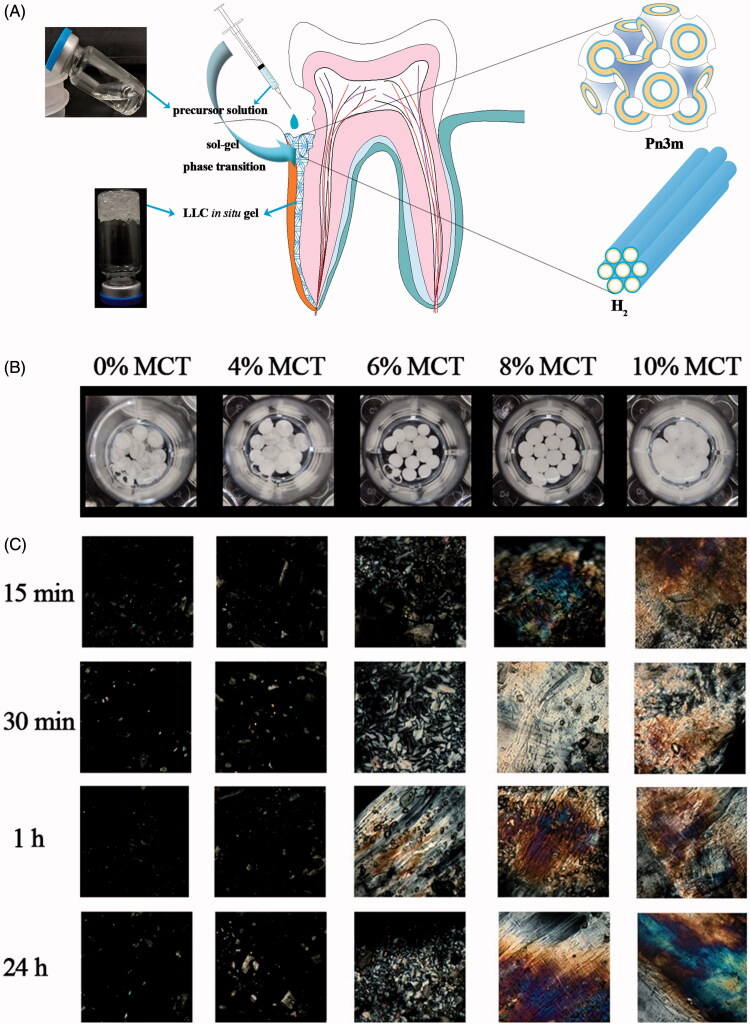
(A) Schematic illustration of the LLC *in situ* forming gel with cubic and hexagonal nanostructures to deliver metronidazole to the periodontal pocket, (B) visualization of the 25% w/w MTZ-loaded LLC gels during drug release under the light view of microscopy, and (C) polarized view of the 25% w/w MTZ-loaded LLC *in situ* gels at various times post-contact of the precursor and excess water at 37 ± 0.5 °C. (Image magnification 220×).

The MTZ-loaded precursor would transform into adhesive gel for firm attachment to the periodontal pockets and sustained release of MTZ. The liquid crystalline phases of the LLC gels during the drug release were examined by CPM at 37 ± 0.5 °C. Figure 1(B) shows the visual appearance of the LLC gels, which were spherical particles. Under the polarized microcopy (Figure 1(C)), the LLC gels without MCT exhibited dark view, indicating the isotropous cubic phase. With 4% MCT content, the birefringence texture started to emerge in the dark background, suggesting mixed phases of hexagonal and cubic phase. The liquid crystalline phase transformed to entire hexagonal phase when the MCT content reached 10%. The phase changes at various time post-hydration of precursors with excess water revealed their dynamic phase transition which was caused by exchange of NMP in the precursors and water in aqueous dissolution medium. No further phase transition was observed after 1 h, indicating the phase equilibrium.

**Figure 2.**
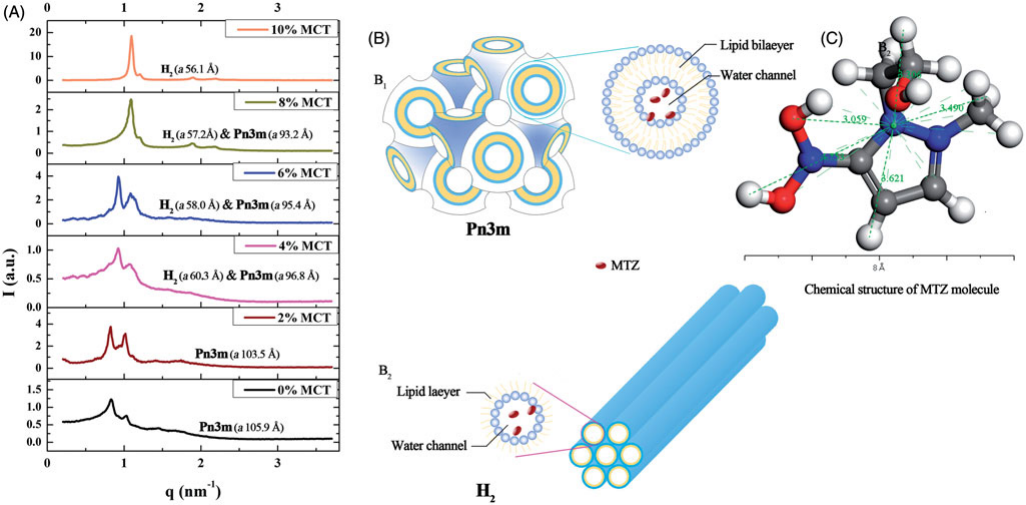
SAXS spectra, liquid crystalline nanostructures, and MTZ structure. (A) Effect of various MCT amounts on the SAXS spectra of the 25% w/w MTZ-loaded LLC gels at 37 ± 0.5 °C, (B) liquid crystalline cell nanostructures of (B_1_) Pn3m nanostructure and (B_2_) H2 nanostructure, (C) the chemical structure and size of MTZ molecule.

The more specific phase identification of crystalline phase was performed by SAXS post-phase equilibrium. The *q* value indicating positions of each Bragg peaks on the SAXS spectra (Figure 2(A)) was noted to identify the phases of the LLC gels containing different MCT contents. Two types of crystalline cells were detected: inverse bicontinuous cubic phase (Pn3m) corresponding to *q* value ratios of $\sqrt{2}:\sqrt{3}:\sqrt{4}:\surd 6$ and inverse hexagonal phase (H_2_) corresponding to *q* value ratios of$\;1:\sqrt{3}:\sqrt{4}:\surd 7$.

The crystalline cell nanostructure of Pn3m consists of a curved lipid bilayer and a bi-continuous water channel extending in three dimensions as a network, and that of H_2_ is composed of several rod-like micelle cylinders lying parallel to each other in a hexagonal array (Figure 2(B)) (Pan et al., 2013). The SAXS spectra revealed that the LLC gel was cubic phase with 0–2% w/w MCT. As the MCT content exceeded 4% w/w of the total weight, the inverse hexagonal phase H_2_ coexisted with inverse cubic phase Pn3m, and a pure H_2_ phase was detected when the MCT content reached 10% w/w. These results were coincident with the optical observations (Figure 1(C)).

Additionally, the lattice parameter *a* was calculated to indicate the size of water channels in the crystalline cell nanostructure (Figure 2(A)). Cubic phase possessed much larger *a* values than hexagonal phase. The *a* values declined in both cubic and hexagonal phase with the increasing amount of MCT. Reduced *a* value revealed marked decrease in the amount of solubilized water and the size of water channels in these liquid crystalline cell nanostructures (Yaghmur et al., 2012a). This could be explained by that increasing addition of hydrophobic MCT into the LLC system increased the volume of lipophilic domain, and the diameter size of the aqueous channels surrounded by the lipid was thus reduced. Reduction in the size of water channels could also be supported by the reduced water absorption due to increasing amount of MCT (Figure S3).

### 3.3. Viscosity and syringe ability of the LLC precursor

**Figure 3.**
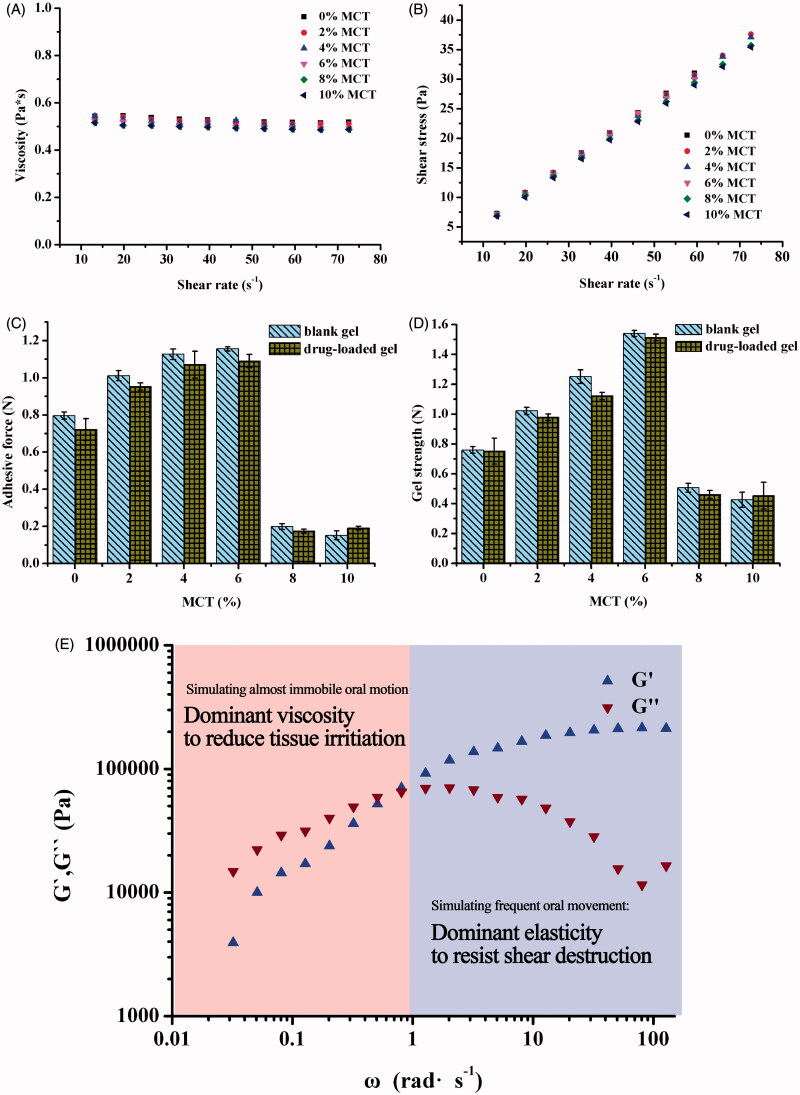
Effect of various MCT amounts on viscosity (A) and shear stress (B) of the 25% MTZ-loaded LLC precursors at various shear rates, 25 ± 0.5 °C. Effect of various MCT amounts and incorporation of MTZ on adhesive force (C) and gel strength (D) of the MTZ-loaded LLC gels evaluated by a texture analyzer at 37 ± 0.5 °C. Effect of changing angular velocity (simulating motion frequency of oral cavity) on the storage modulus *G*′ and the loss modulus *G*″ (E) of the MTZ-loaded LLC gel examined by a rotational rheometer at 37 ± 0.5 °C. All test was conducted in triplicate.

Effect of MCT addition with various amounts on the viscosity and shear stress of the MTZ-loaded LLC precursors at various shear rates was determined to examine the injection feasibility into the periodontal pockets. Figure 3(A) showed that addition of MCT had little influence on the precursor viscosity and all precursors with addition of 0–10% MCT remained low viscosity for penetration to the periodontal pockets. Also, increasing MCT content resulted in slight decrease in the value of shear stress/shear rate (Figure 3(B)), indicating shear thinning property, which favors feasibility of injection.

The precursors had a viscosity lower than 0.6 Pa s (Figure 3(A)), which allowed penetration of the precursor to the minor gaps of periodontal pockets (Bruschi et al., 2007; Dabhi et al., 2010). Then, the injected precursor was transformed into depot drug carrier with custom shape well fitted into the cavity of the periodontal pockets, which would make irritation to the periodontal pockets more tolerable than other depot carriers with intensive plasticity (such as fibers, polymer gels, buccal patches, and microspheres). In addition, the custom shape would increase the contact surface area between the *in situ* gel and the infection sites in the periodontal pockets. The tight contact and increased contact surface area were supposed to facilitate drug absorption of antibiotics and thus enhanced antimicrobial effect.

The complete injection and homogenous drug concentration of the LLC precursor are of critical importance for accurate administration. The ability of total precursor to pass through needles during injection was indicated by syringe ability. Also, dosage accuracy was tested by quantifying the total mass and MTZ concentration of the precursor prior to and post-injection. The syringe test showed that the precursor with low viscosity could be entirely injected, more than 93% w/w (Table S1). The drug content variation after and before syringe test was in the range of 95.5–103.5%, indicating the precursor was homogeneous during injection.

### 3.4. Adhesiveness, gel strength, and viscoelasticity of the LLC gel

#### 3.4.1. Adhesive force and gel strength of the LLC gel

The LLC precursor spontaneously transforms into gel in the presence of water in the oral cavity after being injected into the periodontal pockets, then it needs strong adhesive force and gel strength to assure its firm adhesion to the periodontal pockets and durable resistance to the destruction of mastication motion. Effect of various MCT amounts and MTZ incorporation into the LLC system on adhesive force and gel strength was evaluated by a texture analyzer.

The MCT amounts had the same effects on both the adhesive force and gel strength of the LLC gels (Figure 3(C,D)), which suggested the dependence of mechanical properties of the gels on their liquid crystalline phases and corresponding crystalline cell nanostructures. The increasing MCT amounts improved the adhesive force of the gels (in cubic and hexagonal phase) within 6%, whereas the adhesive force of the gels decreased as the MCT content exceeded 6%, and even dropped below that of MCT-free gel. This could be explained by the crystalline cell nanostructures of cubic and hexagonal phase.

Cubic phase crystalline cell nanostructure (Pn3m) is a sterically stable network consisting of twisty water channels and spreading lipid bilayers, whereas hexagonal phase cell (H_2_) is composed of several individual micelle columns, which array in parallel to each other and could slide in inverse direction. H_2_ structure is more flexible than Pn3m structure (Figure 2(B)). Consequently, the adhesive force in Pn3m phase was higher than that in H_2_ phase as Pn3m phase possessed more intensive nanostructure.

Increasing amounts of MCT reduced the water channel size of the liquid cell nanostructure and thus increased nanostructure compactness, but higher level of MCT content than 6% resulted in phase transition from mixed phases to dominant H_2_ phase. With increasing amount of MCT from 0% to 6%, the adhesive force of the gels gradually increased by the increasing nanostructure compactness, while it dramatically dropped off due to phase transition from mixed phases to dominant H_2_ phase as MCT content exceeded 6%. Taking both crystalline phase types and nanostructure compactness into consideration, the gel containing 6% MCT, in mixed phases of intensive Pn3m and H_2_ phase, showed superior adhesiveness and gel strength, which benefits sustained retention and integrity of the LLC system in the periodontal pockets.

Additionally, the custom shape of the LLC *in situ* forming gel, would achieve intimate contact and intensive adhesion. Actually, the combination of the periodontal pockets and its custom shaped depot drug carrier is kind of male–female mold combination, where the *in situ* gel was incorporated into the curved cavity of the periodontal pockets. It is reasonable to draw a conclusion that this kind of combination in addition to the intensive adhesion of the LLC gel could assume its extended retention at the action sites.

Do et al. reported that the adhesive force of a commercial periodontal Parocline^®^ was no more than 0.1 N, and they increased the maximum adhesive force to 0.4 N by addition of different types and amounts of plasticizers as well as adhesive polymers into their poly(lactic-co-glycolic acid)-based implant (Do et al., 2014). In comparison, the LLC gels containing 4–8% MCT possessed intensive adhesive force far more than 0.4 N (Figure 3(C)), which is sufficient to assure extended duration at action sites, avoiding the drug concentration uncertainty caused by the loss of the drug depot from the periodontal pockets.

In addition to adhesion, the depot drug carrier also requires sufficient mechanical strength to resist the mechanical destruction, such as GCF washing or mastication movement. The variation trend of gel strength and related factors was similar to that of adhesion. Addition of 2–6% MCT increased the gel strength, and reached maximum value of 1.5 N with 6% MCT (Figure 3(D)), higher than the required gel strength of 0.39–1.39 N (Lee et al., 2001; Souza et al., 2014) to resist destruction of mastication movement. The Pn3m nanostructure is a three-dimensional network which could be resistant to stretch and pressure, whereas the H_2_ nanostructure consists of several parallel micellar columns, which could slide from each other. Consequently, the Pn3m phase possessed more intensive gel strength than H_2_ phase. Besides, increasing amount of MCT enhanced nanostructure compactness of the crystalline cell, leading to increase in the gel strength which reached maximum value with 6% MCT, and then dramatically declined due to phase transition from mixed phase to dominant H_2_ phase.

The mechanical properties was key to durable retention of the intra-pocket depot carrier. Relatively high level of adhesion and gel strength indicates that there is no necessity to fix the depot system with other devices because the system itself is strong and sticky enough to remain in the pocket and to cope with physical movement of mastication.

#### 3.4.2. Viscoelasticity of the LLC gel

The viscoelasticity of MCT-loaded LLC gel demonstrated by the storage modulus *G*′ and the loss modulus *G*″ was determined to investigate the response of the gel to various frequency of oral cavity motion (Figure 3(E)). The storage modulus *G*′ indicates elasticity (solid-like property) of the gel, which would resist destruction during mastication movement. The loss modulus *G*″ indicates viscosity (liquid-like property) of the gel. The lower viscosity of the gel, the less irritation it would impose to the surrounding periodontal tissues. The angular frequency, *ω*, indicates the motion frequency of oral cavity. Augmentation in *ω* reduced the loss modulus *G*″ while increased the storage modulus *G*′. When the *ω* exceeded 1 rad s^−1^ and the test simulated the shear of surrounding periodontal tissues to the gel induced by frequent oral cavity movements (such as speaking and mastication), the gel correspondingly exhibited dominant solid-like property (elasticity, *G*′ > *G*″) to resist destruction of the shear force of surrounding periodontal tissues for extended retention and structure integrality of depot carrier. When the *ω* is lower than 1 rad s^−1^ and the test simulated almost immobile motion of the surrounding periodontal tissues, the LLC gel exhibited dominant liquid-like property (viscosity, *G*″ > *G*′) to reduce irritation to inflammatory periodontal pockets. The LLC *in situ* gel possessed excellent viscoelasticity for both superior resilience and compatibility.

### *3.5. In vitro* drug release

Effect of MCT addition with various amounts on cumulative drug release from the 25% w/w MTZ-loaded LLC gels was determined. It was reported that the surface area between the *in situ* formed liquid crystalline phase and the release medium had important influence on the drug release behavior (Yaghmur et al., 2012a). Definite amount of MTZ-loaded precursor was injected into the release medium through a self-modified pipette tips. With slightly higher density than the release medium, the injected precursor precipitated and immerged into the release medium, and transformed into spherical-shape gels with approximately identical surface area (Figure 1(B)).

**Figure 4.**
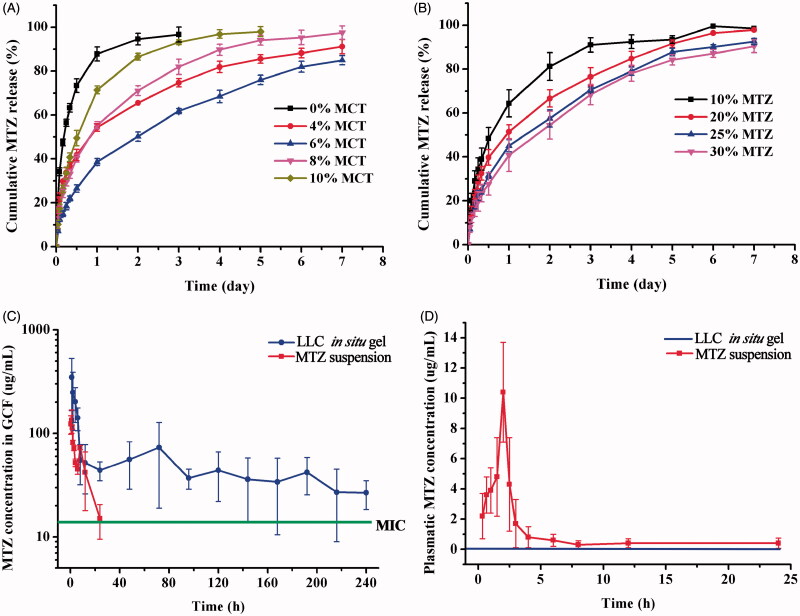
Effect of various MCT amounts (A) and incorporation of different amounts of MTZ (B) on cumulative drug release from the LLC gels at 37 ± 0.5 °C (*n* = 3). *In vivo* study of MTZ-loaded LLC *in situ* gel and MTZ suspension, (C) MTZ concentration in the GCF after local administration of MTZ-loaded LLC *in situ* gel and MTZ suspension, (D) Plasmatic MTZ concentration after administration of MTZ suspension (plasmatic MTZ concentration was under detection after administration of MTZ-loaded LLC *in situ* gel) (*n* = 5).

The results of drug release indicated that addition of MCT sustained drug release from the LLC gels, and the formulations containing 4%, 6%, and 8% MCT illustrated optimal sustained-release over one week (Figure 4(A)), which was desirable for effective treatment of chronic periodontitis (Schwach-Abdellaoui et al., 2000; Bal et al., 2015). The release rate was the smallest when MCT content was 6%. Additionally, increasing drug incorporation content from 10% to 30% reduced the cumulative percentage of released MTZ at each time intervals (Figure 4(B)). Although the drug release rate was faster for the formulation with higher drug loading, there were still higher percentage of remaining drug in the gels because of the increased drug loading.

Also, addition of MCT reduced the initial burst release of MTZ from the LLC gels. The initial release of MTZ from the MCT-free LLC gel in the first 24 h was 91.2% whereas that from the LLC gel containing 6% MCT was only 38.6%. High initial burst release is undesirable for it is the main cause of side effects and antibiotic resistance. Reduced initial burst release is also required to minimize dissipation of total dosage at early stage so as to provide adequate drug dosage for sustained release over one week.

A reasonable hypothesis about drug release mechanism from the LLC gel was proposed by considering several complicated factors, such as variation in the liquid crystalline cell nanostructures, the size of the inner water channels, and the drug release pathways. The model drug MTZ is a hydrophilic molecule which preferred to be incorporated in water channels in the crystalline cell nanostructures. There are significant differences in the crystalline cell nanostructures and water channel size in H_2_ and Pn3m phase. The water channels in H_2_ phase were straight columns independent of each other while that of Pn3m phase was a curved bicontinuous water channel. One water channel of the bicontinuous channel is open to dissolution medium whereas the other one is closed.

Diffusion through the water channels and penetration through the lipid layers were the main release pathways for the drug molecules loaded in the complex nanostructures of the LLC gels (Kunieda et al., 1998). Due to the energy dissipation for penetration, as well as the repulsion between the hydrophilic MTZ and the hydrophobic lipid layer, the hydrophilic MTZ incorporated in the liquid crystalline would be dominantly released by diffusion through water channels.

MTZ molecules were randomly distributed in the water channels as the size of MTZ molecule was smaller than that of water channels in both Pn3m and H_2_ phases. The distances from arbitrary peripheral atoms of the MTZ molecule to its averaged geometry centroid were 3.06–4.75 Å (Figure 2(C)) according to the calculation by the measurement mode in the material studio software (Material Studio 8.0, Accelrys, San Diego, CA). The water channel size in both Pn3m and H_2_ cell was decreased by the increasing MCT addition amounts, for the *a* value of Pn3m cell varying from 105.9 to 93.2 Å and that of H_2_ phase *a* value ranging from 60.3 to 56.1 Å. The *a* values were similar to previous work reported by Yaghmur et al (2007). The water channel size of H_2_ cell was much smaller than that of Pn3m cell, which could also be demonstrated by the water absorption of corresponding precursors (Figure S3).

For pure Pn3m phase without MCT, the water channel size of 105.9 Å was larger than that of MTZ molecules so that the MTZ could freely diffuse from the water channels and was released more rapidly from the pure Pn3m phase than others. The drug release rate from pure H_2_ phase (10% MCT) was slower than that from pure Pn3m phase, even though the drug diffusion pathway through water channel was more direct in the H_2_ phase. This might be attributed to significant decrease in the size of water channel, 56.1 Å for pure H_2_ phase compared to 105.9 Å for Pn3m phase (Figure 2(A)). The same effect of water channel size on the drug release rate was also observed by Fong et al. (2009) and Negrini & Mezzenga (2011).

The MTZ released rate from mixed phases (4–8% MCT) was much smaller than that from pure H_2_ phase or pure Pn3m phase. With MCT amounts from 4% to 8%, the liquid crystalline structure was composed of Pn3m and H_2_ cells. The water channels and lipid layers in the aforementioned cells mutually mixed and twisted together, forming a more complex pathway for drug release. Among them, the formulation containing 6% MCT might possessed approximately identical amount of Pn3m and H_2_ cells, which intermixed and coupled with each other, forming the most compact structures. Therefore, the drug release from the formulation containing 6% MCT was the slowest. The drug release rate from the LLC formulations was: 0% > 10% > 8% > 4% > 6%.

In contrast to a number of reported studies, which draw a simple conclusion that release rate of hydrophilic drug from hexagonal phase is slower than that from cubic phase (Fong et al., 2009; Negrini & Mezzenga, 2011) or otherwise (Yaghmur et al., 2012a), the interesting finding of present study suggests that drug release rates from the LLC formulations consisting of mixed phases of H_2_ and Pn3m phase were slower than any of them. The structural controlled drug release behavior could be achieved by the phase transition and nanostructure modulation of the LLC system.

### *3.6. In vivo* study

*In vivo* studies of the MTZ-loaded LLC gel and MTZ suspension were conducted by determining the MTZ concentration in the GCF and in the blood after administration, which demonstrated the treatment efficacy and systematic side effects, respectively. The LLC *in situ* gel maintained the MTZ concentration above its MIC (Poulet et al., 1999) for over 10 days, without detectable drug in the blood all along (Figure 4(C, D)), which indicated both long-term effective treatment of periodontitis and negligible systematic side effects. Specifically, the LLC *in situ* gel offered high enough initial drug concentration for the clearance of gingival pathogens and then MTZ concentration dramatically dropped down within 24 h, remaining stable in GCF and well above the MIC, which is sufficient to inhibit 90% of bacteria associated with severe periodontitis. The LLC *in situ* gel system comparatively reduced the exposure time of bacteria to unnecessarily high antibiotic concentration, which is the main cause of antibiotic resistance. Also, it is of great importance to modulate the drug release to a relative slow rate following the initial release and avoid excess dissipation of the depot dosage, so that the sustained release could be maintained for long-term treatment of chronic periodontitis. In the control group of MTZ suspension, MTZ concentration in the GCF dramatically fell below the MIC within 24 h, and plasmatic MTZ concentration still remained detectable after 6 h (Figure 4(C,D)).

The *in vivo* pharmacokinetic parameters in the GCF were calculated with WinNonlin software (Phoenix WinNonlin, Certara, Princeton, NJ) and summarized in Table 2. There are significant differences between the *in vivo* parameters of the LLC *in situ* gel and that of control group (*p* < .05), indicating long-term therapeutic effect of the LLC *in situ* gel. Specifically, the *T*_1/2_ and AUC_0–7d_ of the LLC *in situ* gel were respectively improved to 12.9- and 9.3-folds in comparison to the control group, indicating significantly enhanced bioavailability. Apart from the benefit of sustained drug concentration above MIC, the improvement of bioavailability might result from enhanced drug absorption associated with the composite and nanostructures of the LLC system. The LLC system consisted of low molecular weight lipids (GMO and MCT) which are similar to biological lipids, such as glycerides and oleic acid. Therefore, enhanced drug absorption could be achieved by lipid ingestion or exchange between the lipid-based LLC system and surrounding periodontal tissues. Additionally, the lipid ingestion and exchange might be abundant due to the tight contact as well as huge contact area between the periodontal tissues and the custom-shaped LLC *in situ* gel. Another mechanism of the enhanced drug absorption could be explained by the similarity of lipid bilayer in liquid crystalline cell nanostructure and cell membrane. It was reported that the liquid crystalline nanostructure was also found in the human cells (Norlen & Al-Amoudi, 2004), which could induce fusion of the cubic nanostructure between the LLC system and that of surrounding cells, creating pathways for drug penetration and absorption. They are crucial factors to facilitate drug absorption at action sites and thus improve bioavailability.

**Table 2.** *In vivo* parameters of MTZ-loaded LLC *in situ* gel and MTZ suspension in GCF (*x* ± S.D., *n* = 5).

Parameter	LLC *in situ* gel	MTZ suspension
*T*_1/2_ (h)	108.6 ± 24.0*	8.4 ± 3.5
*T*_max_ (h)	1.2 ± 0.4	0.8 ± 0.3
*C*_max_ (mg mL^−1^)	0.37 ± 0.17	0.20 ± 0.08
AUC_0–7d_ (mg mL^−1^)	10.94 ± 1.69*	1.18 ± 0.54
AUC_0–∞_ (mg mL^−1^)	16.88 ± 3.33*	1.45 ± 0.65
*V* (L kg^−1^)	0.25 ± 0.04	0.71 ± 0.28
CL (mL h^−1^)	1.5 ± 0.3*	80.8 ± 27.3
MRT (h^−1^)	95.9 ± 10.4*	9.0 ± 1.0

MTZ: metronidazole; LLC: lyotropic liquid crystalline; GCF: gingival crevice fluid.

*p < .05.

## 4. Conclusions

The intra-pocket LLC system for local treatment of chronic periodontitis achieved significantly enhanced bioavailability and reduced systematic side effects. These results mainly benefited from the rapid solution–gel phase transition and distinct crystalline cell nanostructures of the LLC *in situ* gel.

The LLC system was designed based on the main qualifications for intra-pocket depot system. The LLC *in situ* gel which offered favorable penetration, extended retention in pocket, and sustained drug release exactly satisfied the requirement of intra-pocket depot carrier for local treatment of chronic periodontitis. The sustained release for long-term treatment of periodontitis benefited from the sophisticated nanostructures and variable lattice parameter of crystalline cell nanostructure. The proposed drug release mechanism from the LLC system provides some insights for the development and application of the LLC system in drug delivery but it deserves further investigation and verification, particularly from molecular level.

## Supplementary Material

IDRD_Pan_et_al_Supporting_Information.zip

## References

[CIT0001] Bal MV, Olgun A, Abasli D, et al. (2015). The effect of nonsurgical periodontal treatment on serum and saliva chitotriosidase activities in patients with periodontitis and coronary artery disease. Ther Clin Risk Manag 11:53–8. 25565855 10.2147/TCRM.S76286 PMC4284029

[CIT0002] Barat R, Srinatha A, Pandit JK, et al. (2006). Niridazole biodegradable inserts for local long-term treatment of periodontitis: Possible new life for an orphan drug. Drug Deliv 13:365–73. 16877312 10.1080/10717540500398126

[CIT0003] Bruschi ML, Jones DS, Panzeri H, et al. (2007). Semisolid systems containing propolis for the treatment of periodontal disease: in vitro release kinetics, syringeability, rheological, textural, and mucoadhesive properties. J Pharm Sci 96:2074–89. 17301966 10.1002/jps.20843

[CIT0004] Caboi F, Amico GS, Pitzalis P, et al. (2001). Addition of hydrophilic and lipophilic compounds of biological relevance to the monoolein/water system. I. Phase behavior. Chem Phys Lipids 109:47–62. 11163344 10.1016/s0009-3084(00)00200-0

[CIT0005] Chen X, Wu G, Feng Z, et al. (2016). Advanced biomaterials and their potential applications in the treatment of periodontal disease. Crit Rev Biotechnol 36:760–75. 26004052 10.3109/07388551.2015.1035693

[CIT0006] Da Rocha HaJ, Silva CF, Santiago FL, et al. (2015). Local drug delivery systems in the treatment of periodontitis: a literature review. J Int Acad Periodontol 17:82–90. 26373225

[CIT0007] Dabhi MR, Nagori SA, Gohel MC, et al. (2010). Formulation development of smart gel periodontal drug delivery system for local delivery of chemotherapeutic agents with application of experimental design. Drug Deliv 17:520–31. 20553104 10.3109/10717544.2010.490247

[CIT0008] Do MP, Neut C, Delcourt E, et al. (2014). In situ forming implants for periodontitis treatment with improved adhesive properties. Eur J Pharm Biopharm 88:342–50. 24833006 10.1016/j.ejpb.2014.05.006

[CIT0009] Do MP, Neut C, Metz H, et al. (2015). In-situ forming composite implants for periodontitis treatment: how the formulation determines system performance. Int J Pharm 486:38–51. 25791762 10.1016/j.ijpharm.2015.03.026

[CIT0010] Eke P, Dye B, Wei L, et al. (2012). Prevalence of periodontitis in adults in the United States: 2009 and 2010. J Dent Res 91:914–20. 22935673 10.1177/0022034512457373

[CIT0011] Fong WK, Hanley T, Boyd BJ. (2009). Stimuli responsive liquid crystals provide 'on-demand' drug delivery in vitro and in vivo. J Control Release 135:218–26. 19331865 10.1016/j.jconrel.2009.01.009

[CIT0012] Gong X, Moghaddam MJ, Sagnella SM, et al. (2011). Lyotropic liquid crystalline self-assembly material behavior and nanoparticulate dispersions of a phytanyl pro-drug analogue of capecitabine-A chemotherapy agent. ACS Appl Mater Interfaces 3:1552–61. 21446773 10.1021/am200117u

[CIT0013] Hasturk H, Kantarci A, Ebrahimi N, et al. (2006). Topical H2 antagonist prevents periodontitis in a rabbit model. Infect Immun 74:2402–14. 16552070 10.1128/IAI.74.4.2402-2414.2006 PMC1418940

[CIT0014] Hau H, Rohanizadeh R, Ghadiri M, Chrzanowski W. (2014). A mini-review on novel intraperiodontal pocket drug delivery materials for the treatment of periodontal diseases. Drug Deliv Transl Res 4:295–301. 25786883 10.1007/s13346-013-0171-x

[CIT0015] He J, Zhu X, Qi Z, et al. (2015). Killing dental pathogens using antibacterial graphene oxide. ACS Appl Mater Interfaces 7:5605–11. 25705785 10.1021/acsami.5b01069

[CIT0016] Joshi D, Garg T, Goyal AK, Rath G. (2016). Advanced drug delivery approaches against periodontitis. Drug Deliv 23:363–77. 25005586 10.3109/10717544.2014.935531

[CIT0017] Kelly HM, Deasy PB, Ziaka E, Claffey N. (2004). Formulation and preliminary in vivo dog studies of a novel drug delivery system for the treatment of periodontitis. Int J Pharm 274:167–83. 15072793 10.1016/j.ijpharm.2004.01.019

[CIT0018] Kunieda H, Ozawa K, Huang K-L. (1998). Effect of oil on the surfactant molecular curvatures in liquid crystals. J Phys Chem B 102:831–8.

[CIT0019] Lee B-S, Lee C-C, Wang Y-P, et al. (2016). Controlled-release of tetracycline and lovastatin by poly (d, l-lactide-co-glycolide acid)-chitosan nanoparticles enhances periodontal regeneration in dogs. Int J Nanomed 11:285. 10.2147/IJN.S94270 PMC4723100 26848264

[CIT0020] Lee J, Young S, Kellaway I. (2001). Water quantitatively induces the mucoadhesion of liquid crystalline phases of glyceryl monooleate. J Pharm Pharmacol 53:629. 11370702 10.1211/0022357011775956

[CIT0021] Li XW, Liu WG, Ye GX, et al. (2005). Thermosensitive N-isopropylacrylamide-N-propylacrylamide-vinyl pyrrolidone terpolymers: synthesis, characterization and preliminary application as embolic agents. Biomaterials 26:7002–11. 16024073 10.1016/j.biomaterials.2005.05.094

[CIT0022] Mei L, Xie Y, Jing H, et al. (2017). A novel design for stable self-assembly cubosome precursor-microparticles enhancing dissolution of insoluble drugs. Drug Dev Ind Pharm 43:1239–43. 28276277 10.1080/03639045.2017.1304958

[CIT0023] Mezzenga R, Schurtenberger P, Burbidge A, Michel M. (2005). Understanding foods as soft materials. Nat Mater 4:729–40. 16195765 10.1038/nmat1496

[CIT0024] Nasra MMA, Khiri HM, Hazzah HA, Abdallah OY. (2017). Formulation, in-vitro characterization and clinical evaluation of curcumin in-situ gel for treatment of periodontitis. Drug Deliv 24:133–42. 28156166 10.1080/10717544.2016.1233591 PMC8241198

[CIT0025] Negrini R, Mezzenga R. (2011). pH-responsive lyotropic liquid crystals for controlled drug delivery. Langmuir 27:5296–303. 21452814 10.1021/la200591u

[CIT0026] Nguyen S, Hiorth M. (2015). Advanced drug delivery systems for local treatment of the oral cavity. Ther Deliv 6:595–608. 26001175 10.4155/tde.15.5

[CIT0027] Norlen L, Al-Amoudi A. (2004). Stratum corneum keratin structure, function, and formation: the cubic rod-packing and membrane templating model. J Invest Dermatol 123:715–32. 15373777 10.1111/j.0022-202X.2004.23213.x

[CIT0028] Pan X, Han K, Peng X, et al. (2013). Nanostructured cubosomes as advanced drug delivery system. Curr Pharm Des 19:6290–7. 23470001 10.2174/1381612811319350006

[CIT0029] Phaechamud T, Mahadlek J, Chuenbarn T. (2016). In situ forming gel comprising bleached shellac loaded with antimicrobial drugs for periodontitis treatment. Mater Des 89:294–303.

[CIT0030] Poulet PP, Duffaut D, Lodter JP. (1999). Metronidazole susceptibility testing of anaerobic bacteria associated with periodontal disease. J Clin Periodontol 26:261–3. 10223399 10.1034/j.1600-051x.1999.260411.x

[CIT0031] Qin L, Mei L, Shan Z, et al. (2016). Phytantriol based liquid crystal provide sustained release of anticancer drug as a novel embolic agent. Drug Dev Ind Pharm 42:307–16. 26035332 10.3109/03639045.2015.1052079

[CIT0032] Reise M, Wyrwa R, Muller U, et al. (2012). Release of metronidazole from electrospun poly(L-lactide-co-D/L-lactide) fibers for local periodontitis treatment. Dent Mater 28:179–88. 22226009 10.1016/j.dental.2011.12.006

[CIT0033] Sagalowicz L, Mezzenga R, Leser ME. (2006). Investigating reversed liquid crystalline mesophases. Curr Opin Colloid Interface Sci 11:224–9.

[CIT0034] Saw CLL, Olivo M, Chin WWL, et al. (2007). Superiority of N-methyl pyrrolidone over albumin with hypericin for fluorescence diagnosis of human bladder cancer cells implanted in the chick chorioallantoic membrane model. J Photochem Photobiol B-Biol 86:207–18. 10.1016/j.jphotobiol.2006.10.003 17134910

[CIT0035] Schwach-Abdellaoui K, Vivien-Castioni N, Gurny R. (2000). Local delivery of antimicrobial agents for the treatment of periodontal diseases. Eur J Pharm Biopharm 50:83–99. 10840194 10.1016/s0939-6411(00)00086-2

[CIT0036] Scriven L. (1976). Equilibrium bicontinuous structure. Nature 263:123–5.

[CIT0037] Souza C, Watanabe E, Borgheti-Cardoso LN, et al. (2014). Mucoadhesive system formed by liquid crystals for buccal administration of poly (hexamethylene biguanide) hydrochloride. J Pharm Sci 103:3914–23. 25336429 10.1002/jps.24198

[CIT0038] Srivastava M, Kohli K, Ali M. (2016). Formulation development of novel in situ nanoemulgel (NEG) of ketoprofen for the treatment of periodontitis. Drug Deliv 23:154–66. 24786482 10.3109/10717544.2014.907842

[CIT0039] Van Dyke TE. (2008). The management of inflammation in periodontal disease. J Periodontol 79:1601–8. 18673016 10.1902/jop.2008.080173 PMC2563957

[CIT0040] Wadsater M, Barauskas J, Nylander T, Tiberg F. (2014). Formation of highly structured cubic micellar lipid nanoparticles of soy phosphatidylcholine and glycerol dioleate and their degradation by triacylglycerol lipase. ACS Appl Mater Interfaces 6:7063–9. 24779728 10.1021/am501489e

[CIT0041] Warren DB, Anby MU, Hawley A, Boyd BJ. (2011). Real time evolution of liquid crystalline nanostructure during the digestion of formulation lipids using synchrotron small-angle X-ray scattering. Langmuir 27:9528–34. 21678977 10.1021/la2011937

[CIT0042] Wibroe PP, Azmi IDM, Nilsson C, et al. (2015). Citrem modulates internal nanostructure of glyceryl monooleate dispersions and bypasses complement activation: Towards development of safe tunable intravenous lipid nanocarriers. Nanomed: Nanotechnol Biol Med 11:1909–14. 10.1016/j.nano.2015.08.003 26348655

[CIT0043] Yaghmur A, De Campo L, Sagalowicz L, et al. (2005). Emulsified microemulsions and oil-containing liquid crystalline phases. Langmuir 21:569–77. 15641825 10.1021/la0482711

[CIT0044] Yaghmur A, De Campo L, Salentinig S, et al. (2006). Oil-loaded monolinolein-based particles with confined inverse discontinuous cubic structure (Fd 3 m). Langmuir 22:517–21. 16401095 10.1021/la052109w

[CIT0045] Yaghmur A, Laggner P, Zhang S, Rappolt M. (2007). Tuning curvature and stability of monoolein bilayers by designer lipid-like peptide surfactants. PLoS One 2:e479. 17534429 10.1371/journal.pone.0000479 PMC1868779

[CIT0046] Yaghmur A, Larsen SW, Schmitt M, et al. (2011). In situ characterization of lipidic bupivacaine-loaded formulations. Soft Matter 7:8291–5.

[CIT0047] Yaghmur A, Rappolt M, Ostergaard J, et al. (2012a). Characterization of bupivacaine-loaded formulations based on liquid crystalline phases and microemulsions: the effect of lipid composition. Langmuir 28:2881–9. 22247936 10.1021/la203577v

[CIT0048] Yaghmur A, Sartori B, Rappolt M. (2012b). Self-assembled nanostructures of fully hydrated monoelaidin–elaidic acid and monoelaidin–oleic acid systems. Langmuir 28:10105–19. 22690845 10.1021/la3019716

