# Correction to: Mei et al., An injectable *in s*itu gel with cubic, hexagonal nanostructures for local treatment of chronic periodontitis

**DOI:** 10.1080/10717544.2017.1383014

**Published:** 2017-09-28

Mei L, Huang X, Xie Y, Chen J, Huang Y, Wang B, Wang H, Pan X, Wu C. (2017). An injectable *in s*itu gel with cubic, hexagonal nanostructures for local treatment of chronic periodontitis. *Drug Delivery*. 24:1148–1158. https://doi.org/10.1080/10717544.2017.1359703.

When the above article was first published online, the order of the author was incorrect. The correct order is: ‘Liling Mei, Xintian Huang, Yecheng Xie, Jintian Chen, Ying Huang, Bei Wang, Hui Wang, Xin Pan and Chuanbin Wu’.

This has now been corrected.

