# Supplementary material for: An injectable in situ gel with cubic and hexagonal nanostructures for local treatment of chronic periodontitis
Source: Drug Deliv. 2017 Aug 17;24(1):1148–58. doi: 10.1080/10717544.2017.1359703 (PMC8241103; doi:10.1080/10717544.2017.1359703)
Supplement: IDRD_Pan_et_al_Supporting_Information.zip [file IDRD_A_1359703_SM8388.zip › Supporting Information.docx]

**An Injectable *in Situ* Gel with Cubic and Hexagonal Nanostructures for Local Treatment of Chronic Periodontitis**

*Liling Mei, Yecheng Xie, Xintian Huang, Jintian Chen, Ying Huang, Bei Wang, Hui Wang, Xin Pan*, Chuanbin Wu**

School of Pharmaceutical Sciences, Sun Yat-sen University, Guangzhou 510006, China

* Corresponding Author: Chuanbin Wu, e-mail: chuanbin_wu@126.com;

Xin Pan, e-mail: pxin_1385@163.com.

**Supporting information**

1. Equilibrium solubility of metronidazole in several solvents

To increase the MTZ solubility inside the lyotropic liquid crystalline as well as improve the MTZ loading content in the LLC system, the solubility of MTZ in several solvents, like water, phosphate buffer solution with various pH values (PBS pH2.0, PBS 6.8 and PBS 7.4), ethanol, 1, 2-propylene glycol (PG), dimethylacetamide (DMAC) and N-methyl pyrrolidone (NMP) were tested. Among these tested solvents, NMP and DMAC possessed a superior solubility higher than 200 g·L^-1^(Figure S1). NMP was selected based on high MTZ solubility and the safety for buccal application.





Figure S1. Solubility of metronidazole in several solvents at 25 ± 0.5°C (*n* = 3).

1. Liquid crystalline phase transition induced by addition of MCT

To define the MCT content, the liquid crystalline phase transition induced by addition of MCT and temperature variation was investigated from room temperature (25°C) to above the body temperature (40°C).





Figure S2. Effect of various MCT amounts and change of temperature on crystalline phases of blank LLC *in situ* gels identified by the cross-polarized microscopy equipped with a thermo-stage.

The influence of MCT addition on the crystalline phase of the LLC *in situ* gels revealed that addition of MCT did not affect the cubic phase of LLC *in situ* gel within 2% w/w. The hexagonal phase emerged when the MCT content exceeded 3% at 30℃ and above, and the crystalline phase transformed into completely hexagonal phase with 8-11% MCT but gradually changed to inverse micellar as the MCT content exceeded the 11%.

Also, increasing temperature facilitated the cubic-hexagonal-micellar phase transition, that is, the higher temperature, the lower content of MCT was required to induce phase transition. Addition amount of MCT for further investigation ranged from 0% to10% to obtain cubic phase and hexagonal phase, which were reported to retard the drug release via incorporation of drug into their complex nanostructure networks.

1. Syringeability of the LLC precursor

The syringeability of the LLC precursor containing 52.6% GMO, 13.2% NMP, 4.2% MTZ and 30% MCT w/w, was evaluated by passing a weighed amount of the precursor preparation through a 1 mL syringe with 18, 20, 22 and 24-gauge needles, respectively. The preparation after injection was collected and weighed. In addition, the drug concentrations in the precursor prior and post syringeability test were also determined to evaluate the content uniformity.

The ability of total precursor to pass through needles during injection was indicated by syringeability. Also, dosage accuracy was tested by quantifying the total masses and MTZ concentrations of the precursor prior and post injection

Table S1. Syringe ability of the optimal LLC precursor at 25 ± 0.5°C (n=3）

| Variation (%) | 18 G | 20 G | 22 G | 24 G |
| --- | --- | --- | --- | --- |
| *M*_po._/*M*_pr._ | 93.14 ± 1.24 | 93.21 ±1.06 | 96.26 ± 0.73 | 94.36 ± 0.69 |
| *C*_po._/*C*_pr._ | 103.22 ± 0.82 | 99.56 ± 0.75 | 103.54 ± 0.77 | 100.88 ± 0.63 |

*M*_po._ and *M*_pr._ indicated the total masses of the precursor post and prior syringe test, respectively; *C*_po._ and *C*_pr._ indicated the drug concentrations of the precursor post and prior syringe test, respectively.

The complete injection and homogenous drug concentration are of critical importance for accurate administration. The syringeability test showed that the precursor with low viscosity could be entirely injected, more than 93% w/w (Table S2). The drug content variation after and before syringe test was in the range of 95.5~103.5%, indicating the precursor was homogeneous during injection.

1. Water absorption

In order to explore the effect of various MCT amounts on the water channel size, the water absorption was determined. Specifically, 5 mL 37 ± 0.5°C thermostatic water was added to a 10 mL vial (weight M_0_ g) and the total weight of the vial and added water was recorded as M_1_ g. Then, 0.2 mL precursor solution was added to the vial by a modified pipette tip, with the weight of whole system recorded as M_2_ g. The vials were sealed immediately and followed by shaking at 37 ± 0.5℃, 100 rpm. After 1, 2, 4, 6, 8 and 12 h, the excess water was removed and the weight of vial without free water was recorded as M_t_. Finally, another 5 mL water was added to the vials and kept shaking till the next sampling interval. Water absorption $\text{M}_{\text{ab}}$ was calculated by equation (S1):

$\text{M}_{\text{ab}}\text{\% }\text{=(}\frac{\text{M}_{\text{t}}\text{-}\text{M}_{\text{0}}}{\text{M}_{\text{2}}\text{-}\text{M}_{\text{1}}}\text{-1)×}\text{100\%}$ (S1)

M_t_-M_0_ indicates the total weight of the *in situ* gel after absorbing water; M_2_-M_1_ indicates the weight of added precursor solution.





Figure S3. Effect of various MCT amounts on the water absorption of the LLC precursor at 37 ± 0.5°C (*n* = 3).

The water absorption of LLC precursor for the fully hydration decreased by increasing MCT content (Figure S3). The hydrophobic to hydrophilic domain ratio was notably enlarged because the MCT insert in the lipid domain resulting in larger hydrophobic volume whereas the reduced water absorption minimized the hydrophilic domain, namely the water channel size. Water absorption reached equilibrium within 2 h.

1. Video S1, video of the solution-gel phase transition upon the contact of the precursor solution and excess water.
2. Video S2, video of the phase equilibrium between hexagonal and cubic phase record by the cross-polarized microscopy equipped with a camera. The anisotropic hexagonal phase was indicated by the birefringence texture, while the cubic phase revealed dark view under the polarized view due to its isotropic property. The phase transition was caused by water diffusion within the LLC system, leading to phase equilibrium.
3. Methods for *in vivo* sampling and HPLC analysis

***In vivo* sampling**. 1 mL blood sample was collected from auricular vein of treated rabbits with heparin vials at each predetermined time post administration. The samples were centrifuged at 5000 rpm for 5 min and the resulting supernatants were stored at -20°C for quantification of plasma MTZ concentration

Sterile orthodontic points were utilized for the sampling of gingival crevicular fluid (GCF), which were inserted into the bottom of each periodontal pocket and kept in place for 10 s. The sterile orthodontic points were placed in the weighed vials and then the vials were accurately reweighed by a microbalance to calculate the amount of GCF sample obtained by the orthodontic points. The obtained GCF samples were extracted and diluted by the mobile phase consisting of acetonitrile/water (20/80, v/v) for further HPLC analysis.

**HPLC analysis** The HPLC system (CBM-10Avp plus, Shimadzu, Japan) for MTZ quantification was equipped with a C_18_ column (5 μm, 6.0 × 250 mm, Gemini, phenomenex) at 37 ± 0.5°C. The mobile phase for analysis of *in vitro* dissolution and *in vivo* test consisted of methanol/water (10/90, v/v) and acetonitrile/water (20/80, v/v), respectively. The injection volume was 20 μL and the flow rate was set at 1 mL·min^-1^. The column effluent was detected at a wavelength of 315 nm.
